# Nontraumatic bladder rupture: The images before and after

**DOI:** 10.1002/ccr3.4312

**Published:** 2021-06-10

**Authors:** Mitsumasa Okano, Toshihiko Oshita, Kazunori Otsui, Kazuhiko Sakaguchi

**Affiliations:** ^1^ Division of General Internal Medicine Department of Internal Medicine Kobe University Graduate School of Medicine Kobe Japan

**Keywords:** acute cystitis, bladder diverticulum, bladder rupture, CT scan, retrograde cystography

## Abstract

We should consider the complication of bladder rupture for patients with worsened abdominal pain and inability to pass urine following acute cystitis. A CT scan is a useful first‐line modality when evaluating for a suspected bladder rupture.

## Graphical abstract

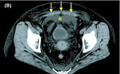

## 1 INTRODUCTION

**FIGURE 1:**
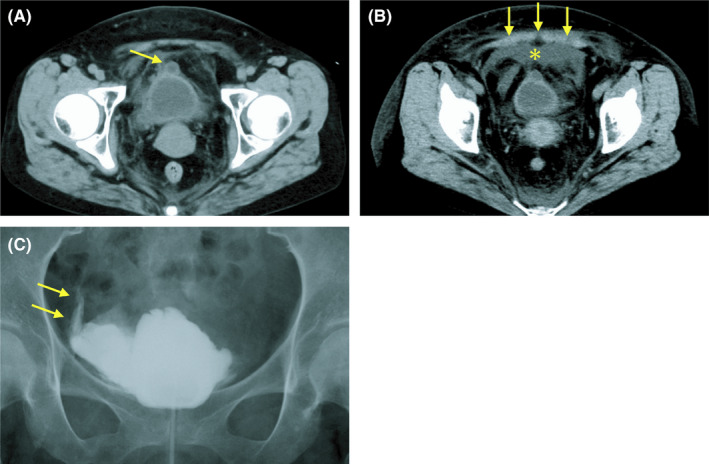
A, Initial CT scan showed bladder diverticulum (arrow) and thickened bladder wall with stranding in the surrounding fat. B, Repeated CT scan showed fluid collection in anteroventral portion of the bladder (asterisk) with thickening of pelvic peritoneum (arrows). C, Retrograde cystography showed contrast material moving outside the bladder (arrows)

A 50‐year‐old woman, taking prednisolone for 20 years to her autoimmune hepatitis, presented with suprapubic pain during urination. She was diagnosed with acute cystitis based on pyuria and bacteriuria. Her initial CT scan showed a bladder diverticulum (Figure 1A). The next day, she was admitted to our hospital because of worsened symptom despite antibiotic therapy. Physical examination revealed suprapubic tenderness with guarding. Laboratory findings showed elevated inflammation markers and serum creatinine level. Repeated CT scan showed the fluid collection in the anteroventral portion of the bladder with thickening of pelvic peritoneum (Figure 1B). Her retrograde cystography revealed contrast material moving outside the bladder into extraperitoneal space (Figure 1C). The perforation site of the diverticulum was not evident by cystoscopy. She was diagnosed with extraperitoneal bladder rupture, which led to pelvic peritonitis and pseudo‐renal failure due to creatinine reabsorption across the peritoneal membrane. The insertion of a urethral catheter improved her symptom dramatically. In addition, urapidil was initiated to the neurogenic bladder as a suspected cause of bladder outlet obstruction and diverticulum.

Pathological bladder‐wall fragility and increased intrabladder pressure are recognized as risk factors for nontraumatic bladder rupture. In our case, sequential CT images suggested that distended bladder caused by acute cystitis provoked bladder rupture on the background of bladder‐wall fragility due to long‐term steroid use^1^ and/or diverticulum.^2^

## CONFLICT OF INTEREST

None declared.

## AUTHOR CONTRIBUTIONS

MO: drafted the manuscript. TO, KO, and KS: supervised the manuscript. All authors read and approved the final manuscript.

## Data Availability

The data that support the findings of this study are available from the corresponding author upon reasonable request.

## References

[1] Ribeiro GS , Souza DB , Cortez CM , Silva D , Costa WS , Sampaio FJ . Effects of prepubertal corticosterone treatment on urinary bladder. Acta Cir Bras. 2014;29(Suppl 3):55‐59. 10.1590/s0102-86502014001700011 25351158

[2] Kodama K , Takase Y , Saito K . Extraperitoneal rupture of a bladder diverticulum and the role of multidetector computed tomography cystography. Urol Case Rep. 2016;9:30‐32. 27656418 10.1016/j.eucr.2016.08.005 PMC5030335

